# Eco-friendly second-derivative synchronous fluorescence method for the determination of empagliflozin and sitagliptin in tablets and plasma samples

**DOI:** 10.1038/s41598-026-53178-z

**Published:** 2026-05-20

**Authors:** Ahmad A. A. Mohamad, Ahmed A. Almrasy, Ahmed H. Abdelazim, Ahmed S. Taha

**Affiliations:** Pharmaceutical Analytical Chemistry Department, Faculty of Pharmacy, Al- Azhar University, Cairo, 11751 Egypt

**Keywords:** Empagliflozin, Sitagliptin, Fluorimetry analysis, Second derivative synchronous, And Eco-friendly analysis, Biochemistry, Chemistry

## Abstract

Empagliflozin and sitagliptin are commonly co-administered to manage type 2 diabetes mellitus due to their complementary mechanisms of action on renal glucose reabsorption and insulin secretion, respectively. This synergistic effect enhances glycemic control with a minimal risk of hypoglycemia. In this study, a sensitive, simple, rapid, and selective spectrofluorimetric method was developed for the simultaneous determination of empagliflozin in the presence of sitagliptin in pharmaceutical formulations and spiked human plasma. Both drugs exhibit native fluorescence; however, their emission spectra significantly overlap, complicating their simultaneous analysis with conventional spectrofluorimetric techniques. To overcome this limitation, second-derivative synchronous spectrofluorimetry was applied. Measurements were performed within an optimized wavelength interval (Δλ = 60 nm), enabling selective determination of sitagliptin at 393 nm and empagliflozin at 310 nm without mutual interference. Experimental parameters affecting fluorescence intensity, including solvent type, pH, and Δλ, were carefully optimized to achieve maximum sensitivity and selectivity. The proposed method demonstrated excellent analytical performance, with limits of detection of 0.031 µg/mL and 0.011 µg/mL, and limits of quantification of 0.093 µg/mL and 0.033 µg/mL for sitagliptin and empagliflozin, respectively. Good linearity was observed over concentration ranges of 0.1–4 µg/mL for sitagliptin and 0.04–1.2 µg/mL for empagliflozin. The method was successfully applied to spiked human plasma samples and validated in accordance with ICH guidelines, demonstrating satisfactory accuracy, precision, robustness, and selectivity. A statistical comparison with a reported method showed no significant difference in performance. The environmental sustainability of the proposed method was comprehensively evaluated using several green analytical chemistry assessment tools, including the Carbon Footprint Reduction Index (CaFRI), Eco-Scale, AGREEprep, CompMoGAPI, and NEMI. In addition, the Blue Applicability Grade Index (BAGI) was employed to assess the method’s blueness, reflecting its practical applicability. Furthermore, the RGB 12 algorithm was applied to evaluate the method’s overall whiteness by integrating its environmental impact (greenness), analytical performance (redness), and practical efficiency (blueness). The obtained results consistently confirmed the excellent eco-friendly profile of the proposed method, demonstrating minimal environmental impact, reduced solvent consumption, strong compliance with green analytical chemistry principles, and high overall performance.

**Supplementary Information:**

The online version contains supplementary material available at 10.1038/s41598-026-53178-z.

## Introduction

In the twenty-first century, diabetes mellitus emerged as a major global health concern^1–3^. The increasing prevalence of type 2 diabetes mellitus is associated with serious complications, including cardiovascular disorders, nephropathy, neuropathy, and retinopathy. Effective glycemic control is therefore essential to reduce the risk of these complications^4,5^, and combination therapy is often required to achieve optimal therapeutic outcomes.

Empagliflozin (EMP) and sitagliptin (SIT) are widely co-formulated in fixed-dose combinations for the management of type 2 diabetes mellitus. EMP, a selective sodium-glucose co-transporter 2 inhibitor, lowers blood glucose levels by reducing renal glucose reabsorption and enhancing urinary glucose excretion^6–8^. In contrast, SIT, a dipeptidyl peptidase-4 inhibitor, improves glycemic control by enhancing incretin activity, thereby stimulating insulin secretion and suppressing glucagon release in a glucose-dependent manner^9,10^. The complementary mechanisms of these drugs result in superior glycemic control compared to monotherapy.

Several analytical techniques have been reported for the determination of EMP, including UV spectrophotometry^11,12^, fluorimetry^13–20^, high-performance liquid chromatography (HPLC)^21–28^, and electrochemical methods^29^. Similarly, SIT has been determined using various techniques, including HPLC^30–34^, UV spectrophotometry^35–39^, fluorimetry^40–44^, and electrochemical methods^45–47^. However, only a limited number of methods have been reported for the simultaneous determination of these compounds in combined dosage forms, mainly HPLC^48^ and a simultaneous-equation UV spectroscopic method^49^. Despite the high sensitivity and selectivity of chromatographic methods, their routine application is often constrained by high cost, significant solvent consumption, and relatively time-consuming procedures.

Spectrofluorimetry offers an attractive alternative due to its high sensitivity, simplicity, cost-effectiveness, and environmental compatibility^50–57^. Nevertheless, the substantial overlap in the native fluorescence spectra of EMP and SIT hinders their direct simultaneous determination. This limitation can be effectively overcome by applying synchronous spectrofluorimetry coupled with derivative processing, thereby enhancing spectral resolution and minimizing spectral overlap.

In this study, a rapid, sensitive, and environmentally friendly second-derivative synchronous spectrofluorimetric method was developed for the simultaneous determination of EMP and SIT in pharmaceutical formulations and spiked human plasma. The method is based on measuring second-derivative synchronous fluorescence amplitudes at Δλ = 60 nm, enabling selective determination of EMP at 310 nm and SIT at 393 nm without mutual interference. Key experimental parameters were optimized to achieve maximum sensitivity and selectivity.

**Fig. 1:**
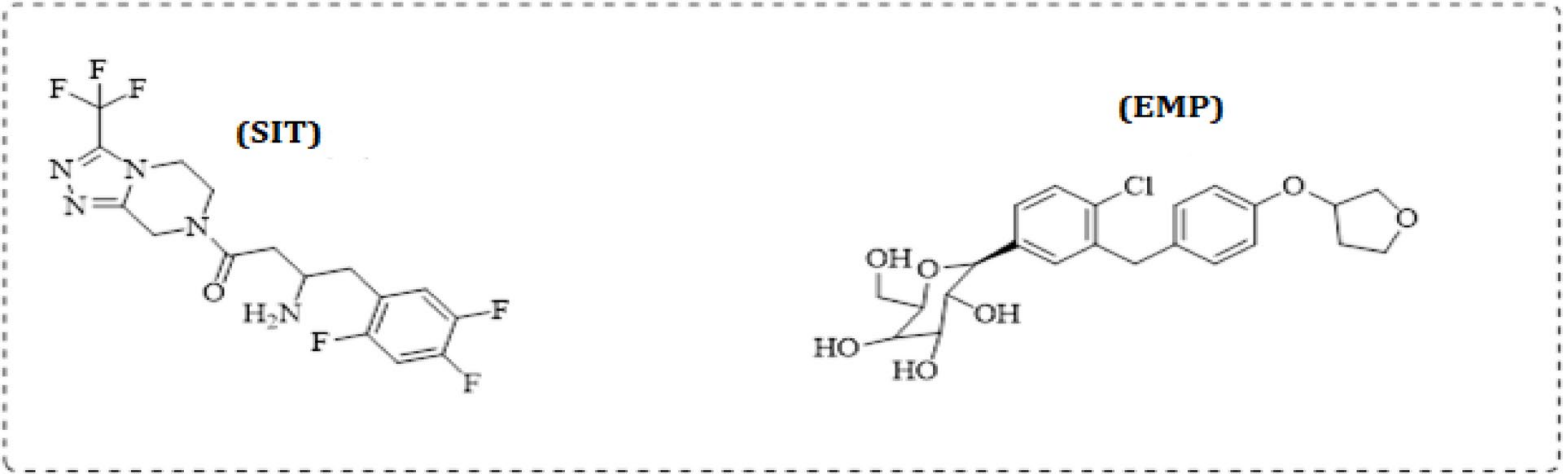
Structural formula for SIT and EMP.

The proposed method exhibited low limits of detection and quantification, with linear responses over 0.04–1.2 µg/mL for EMP and 0.1–4 µg/mL for SIT. It was successfully applied to spiked human plasma and validated in accordance with ICH guidelines^58^, demonstrating satisfactory accuracy, precision, and reliability in pharmaceutical and biological matrices. The environmental sustainability of the proposed method was evaluated using multiple green analytical chemistry assessment tools, including AGREEprep^59^, NEMI^60^, Complex MoGAPI^61^, the Eco-Scale^62^, and CaFRI^63^. In addition, the RGB 12 model was applied to assess the method’s whiteness, integrating green (environmental impact), red (analytical performance), and blue (practical applicability) criteria^64,65^. The Blue Applicability Grade Index (BAGI) was also used to evaluate the method’s practical applicability^66^. The obtained results confirmed the excellent eco-friendly profile of the proposed method, demonstrating minimal solvent consumption, reduced environmental impact, and high overall sustainability without compromising analytical performance. Figure 1.

## Experimental

### Materials

#### Pure samples and pharmaceutical tablets

Pure empagliflozin (99.89% purity) was supplied by DPK Pharmaceuticals, Al Obour City, Egypt. Pure sitagliptin (99.73% purity) was obtained from Hikma Pharmaceuticals Co., 6th of October City, Giza, Egypt. The purity of both standards was confirmed in accordance with the manufacturers’ certificates of analysis. Gliempaflozin SITA 25/100 tablets, B. No EMV250616C, manufactured by Steris Healthcare, India, was listed as having 100 mg of sitagliptin and 25 mg of empagliflozin. The tablets were purchased in India from a local market.

#### Chemicals and reagents

All the solvents and reagents used in the procedure were of analytical grade, and the water used was freshly distilled. Sigma-Aldrich (Germany) provided analytical-grade 1-propanol, ethanol, acetonitrile, and methanol (98.8%). Among the chemical reagents provided by El-Nasr Company (Egypt) were glacial acetic acid, boric acid, sodium hydroxide, sodium acetate, monobasic potassium phosphate, methylcellulose (MC, 0.5% aqueous solution), Tween 80 (0.5% aqueous solution), and sodium dodecyl sulfate (SDS, 0.5% aqueous solution). Buffers for different pH ranges were prepared in accordance with USP regulations^67^.

##### Human plasma

The Blood Bank of Al-Malik Al-Saleh Hospital kindly supplied human plasma samples. The frozen plasma samples were carefully thawed at room temperature before use in preparing spiked samples for method validation.

##### Apparatus

The Jasco FP-6200 Spectrofluorometer (Tokyo, Japan) was used for all measurements, with Jasco Spectra Manager software for spectral data processing. A 1-cm quartz cell was used. The excitation and emission slit widths were adjusted to 5 nm, while the monochromator’s effective spectral bandwidth was 10 nm under the selected configuration. The sensitivity of the measurements was set to medium. The pH was measured using a calibrated Jenway 3510 pH meter (Staffordshire, UK). Weighing was carried out using a Precisa 125 A analytical balance (Switzerland).

Plasma samples were prepared using a high-speed centrifuge (Universal 320, Hettich Zentrifugen, Germany) for phase separation. Mixing was performed with a vortex mixer (Vortex Genius 3, IKA, Germany). Solvent evaporation under reduced pressure was carried out using a rotary evaporator (Rotavapor R-300, Buchi, Switzerland).

##### Standard and working solutions

Stock standard solutions of SIT and EMP (1 mg/ml) were prepared separately by dissolving 100 mg of each drug in methanol and completing to volume in 100 ml volumetric flasks.

## Procedures

### Construction of the calibration curve

Aliquots of standard working solutions of SIT (1–40 µg/ml) and EMP (0.4–12 µg/ml) were accurately transferred into a series of 10-ml volumetric flasks. To each flask, 1.0 ml of 0.50 M acetic acid and 1.0 ml of acetate buffer (0.1 M, pH 4.0) were added, providing an acidic medium suitable for both analytes. This condition enhanced SIT’s native fluorescence by suppressing photoinduced electron transfer. The volume was then adjusted to the mark with methanol, selected for its strong solubilizing ability, protein-precipitating capacity, and favorable environmental profile. Second-derivative synchronous fluorescence spectra were recorded at a constant wavelength difference (Δλ) of 60 nm. The excitation and emission slit widths were both adjusted to 5 nm, and the scanning rate was maintained at 600 nm min⁻¹. Spectral data were acquired using Jasco Spectra Manager software, applying a filter size of 20 and a wavelength interval of 1 nm, and subsequently transformed into second-derivative spectra.

The amplitudes of the second-derivative synchronous spectra were measured at 310 nm for EMP and at 393 nm for SIT. Each drug exhibited a well-defined peak without interference from the other component, indicating good selectivity. Calibration curves were constructed by plotting measured peak amplitudes against corresponding drug concentrations, and the associated regression equations were derived. All procedures were performed in accordance with the relevant analytical guidelines.

### Analysis of Lab-prepared mixture

To obtain final concentrations reflecting the pharmaceutical combination ratio (1:4), appropriate aliquots of EMP and SIT standard stock solutions were transferred into 10 ml volumetric flasks and mixed thoroughly. Subsequently, 1.0 ml of 0.50 M acetic acid and 1.0 ml of acetate buffer (0.1 M, pH 4.0) were added, and the volume was completed to the mark with methanol.

The prepared mixtures were analyzed following the procedure described in the Construction of Calibration Graphs section. The concentrations of EMP and SIT were calculated using the corresponding regression equations. Additionally, percentage recoveries were determined to evaluate the accuracy, precision, and selectivity of the proposed method.

### Analysis of pharmaceutical tablets

Ten tablets of Gliempaflozin^®^ SITA 25/100, each labeled to contain 100 mg of SIT and 25 mg of EMP, were accurately weighed and finely powdered to obtain a homogeneous mixture. An amount of powder equivalent to one tablet was transferred into a 100 ml volumetric flask containing 50 ml of methanol. The mixture was shaken vigorously for 20 min to ensure complete extraction of both active constituents, then filtered, and the volume was completed to the mark with methanol. Appropriate aliquots of the filtrate were further diluted with methanol to prepare five samples within the proposed concentration range. The general procedure described under the Construction of Calibration Graphs section was then applied. The nominal contents of SIT and EMP were determined using the corresponding regression equations.

#### Procedure for spiked human plasma

A series of centrifuge tubes was prepared by transferring 1 ml aliquots of human plasma, spiked with appropriate amounts of SIT and EMP standard working solutions, to achieve final concentrations within the established linearity ranges. Protein precipitation was induced by adding methanol to each tube up to a total volume of 2.0 ml, followed by vortex mixing for 30 s to ensure efficient extraction and disruption of plasma protein binding. The samples were then centrifuged at 3600 rpm for 20 min to achieve clear phase separation. The supernatants were carefully separated and evaporated under reduced pressure using a rotary evaporator.

The resulting dry residues were quantitatively reconstituted in methanol and transferred into 10-ml volumetric flasks. At this stage, 1.0 ml of 0.50 M acetic acid and 1.0 ml of acetate buffer (0.1 M, pH 4.0) were added, and the solutions were diluted to volume with methanol to ensure consistency with the optimized acidic conditions employed in the general procedure and calibration studies.

Blank plasma samples were prepared and processed in parallel following the same procedure to evaluate potential interference from endogenous plasma components and to confirm method selectivity. The final solutions were analyzed according to the procedure described under Construction of Calibration Graphs, and the concentrations of SIT and EMP were determined using the corresponding regression equations.

## Results and discussion

### Spectral characteristics

Synchronous fluorescence spectroscopy (SFS) is a powerful technique for resolving broad, highly overlapping spectra commonly encountered in multicomponent systems, as it produces narrower, more structured spectral bands. Despite this improvement, the resolution achieved by SFS alone is often insufficient to fully discriminate between closely overlapping analytes. Therefore, integrating synchronous fluorescence spectroscopy with derivative techniques has been proposed to further enhance spectral resolution.

**Fig. 2:**
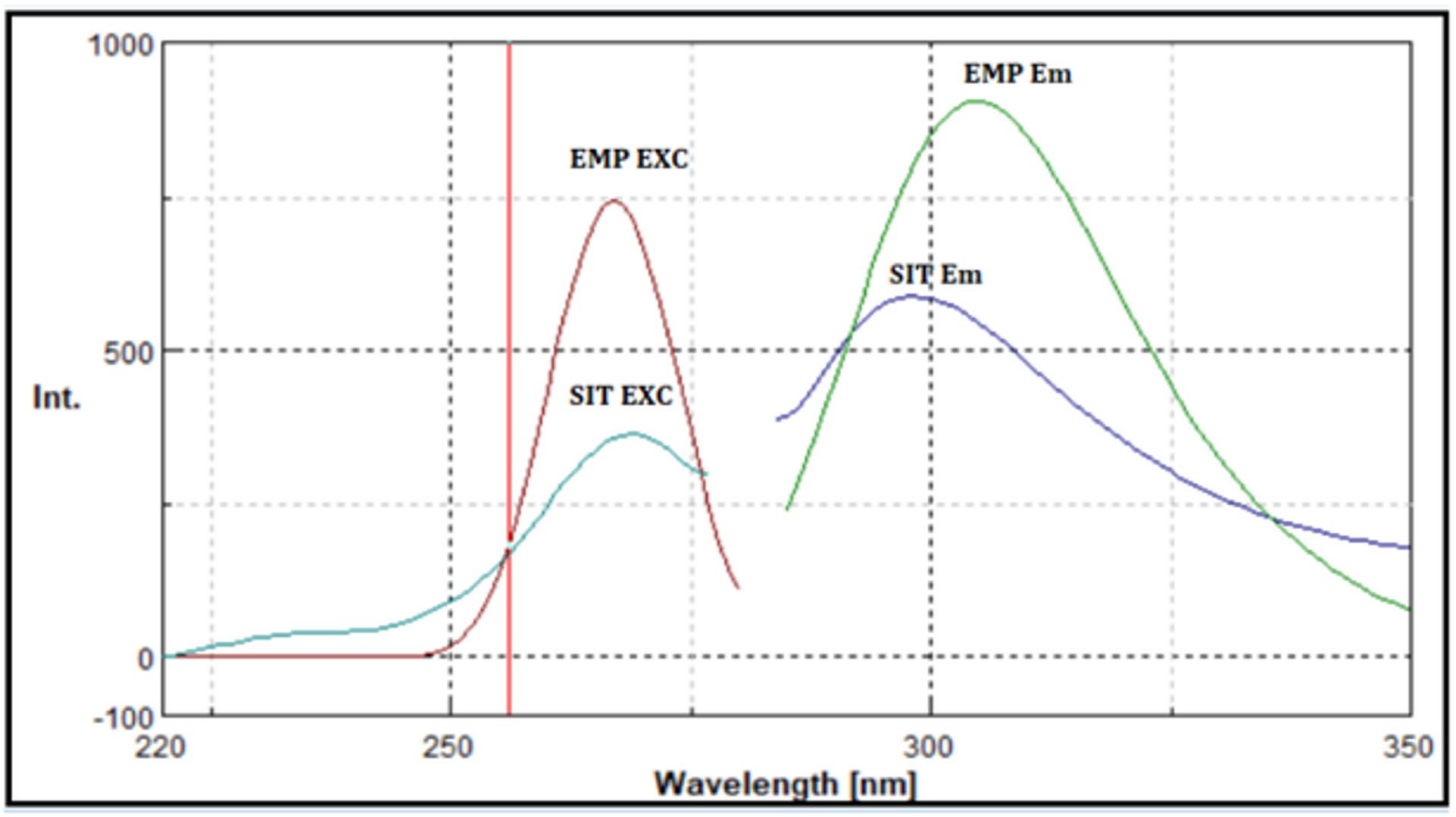
Demonstrates the excitation and emission spectra of EMP and SIT.

**Fig. 3:**
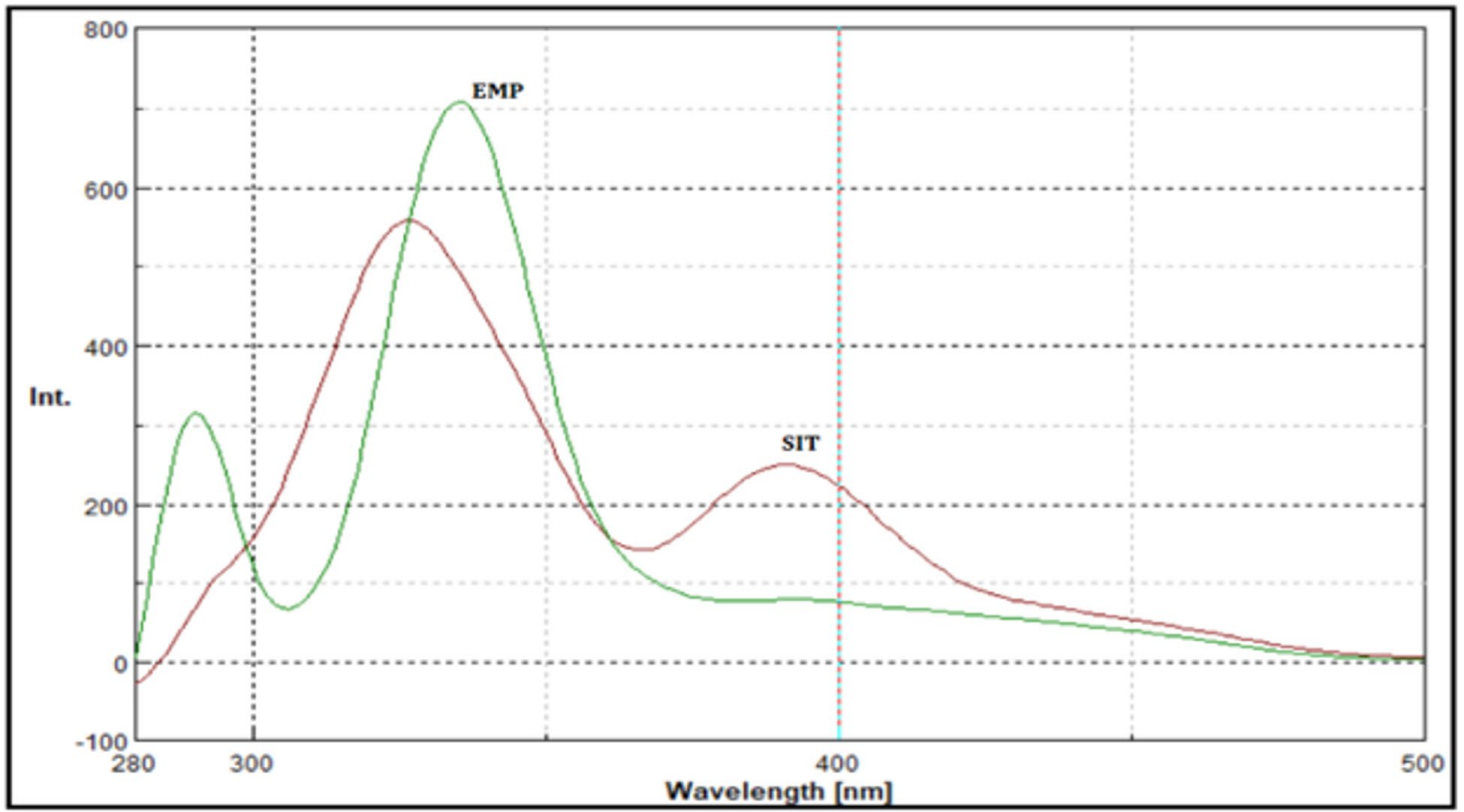
Shows the synchronous fluorescence spectra of EMP (0.9 µg/ml) and SIT (2.8 µg/ml) at Δ = 60 nm.

In the present study, the native excitation and emission spectra of EMP and SIT exhibited pronounced spectral overlap, which hindered their direct simultaneous determination (Fig. 2). Application of synchronous fluorescence spectroscopy at Δλ = 60 nm resulted in partial band narrowing and improved spectral definition (Fig. 3); however, complete resolution between the two analytes was not achieved.

**Fig. 4:**
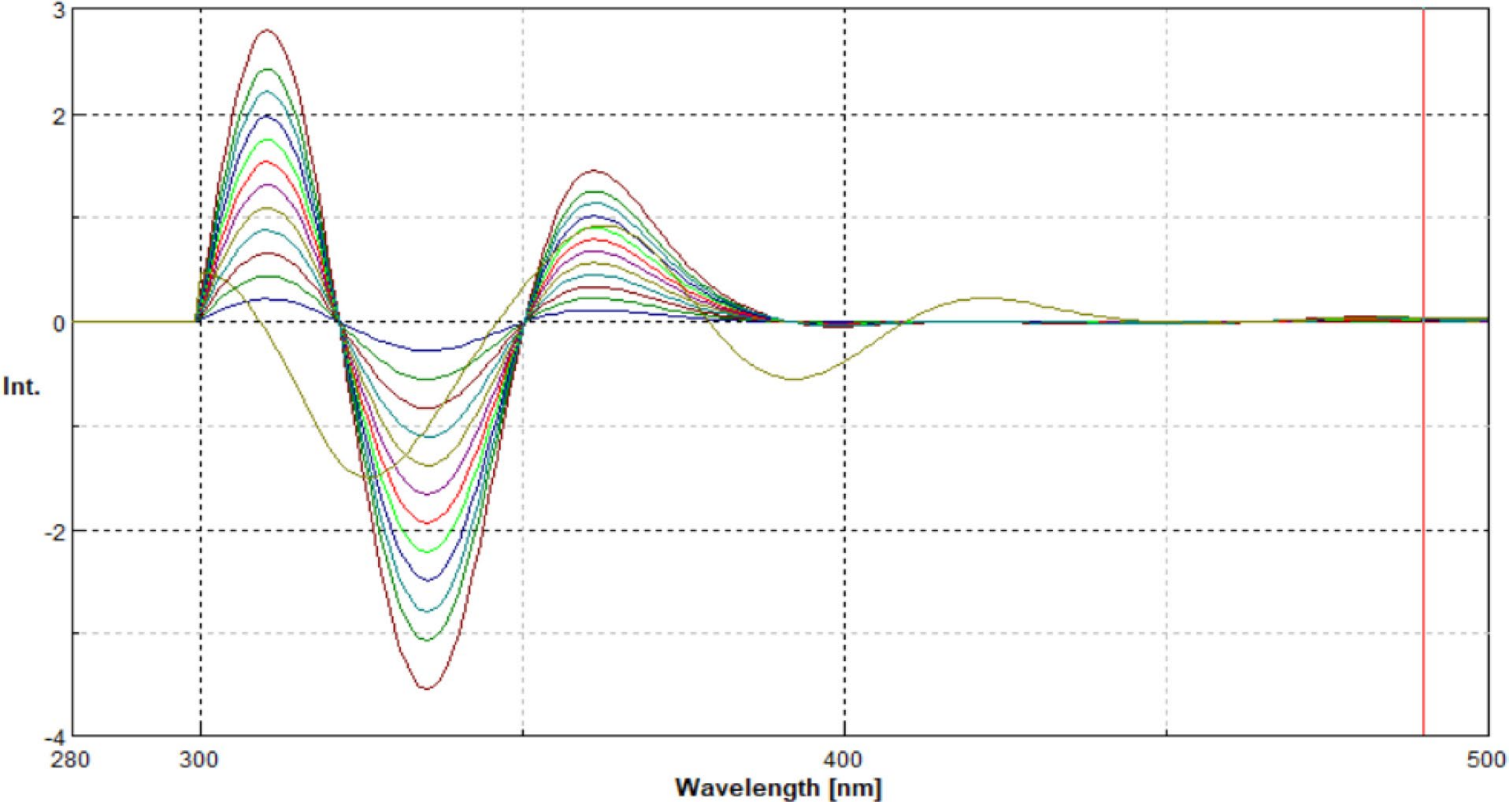
Presents the second-derivative synchronous fluorescence spectra of EMP (0.04–1.2 µg/ml) and SIT (3.2 µg/ml) at 310 nm, at Δ = 60 nm.

**Fig. 5:**
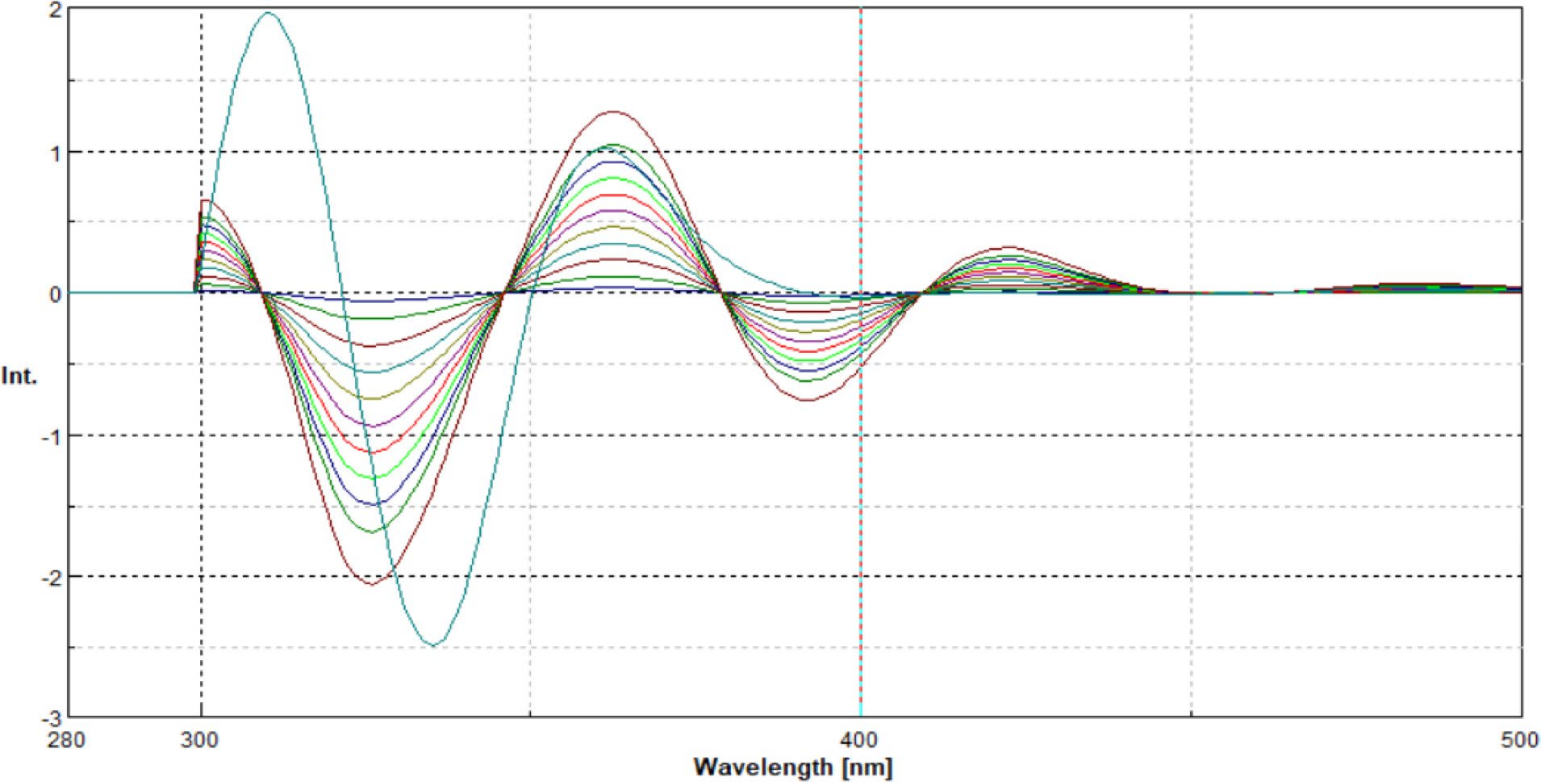
Presents the second-derivative synchronous fluorescence spectra of SIT (0.1–4 µg/mL) and EMP (0.9 µg/mL) at 393 nm, at Δ = 60 nm.

To overcome this limitation, second-derivative processing of the synchronous spectra was employed. This approach markedly enhanced spectral resolution by minimizing overlapping contributions and amplifying subtle spectral differences. As illustrated in Figs. 4 and 5, well-defined, distinct peaks were observed at 310 nm for EMP and at 393 nm for SIT, enabling their selective determination without mutual interference.

**Fig. 6:**
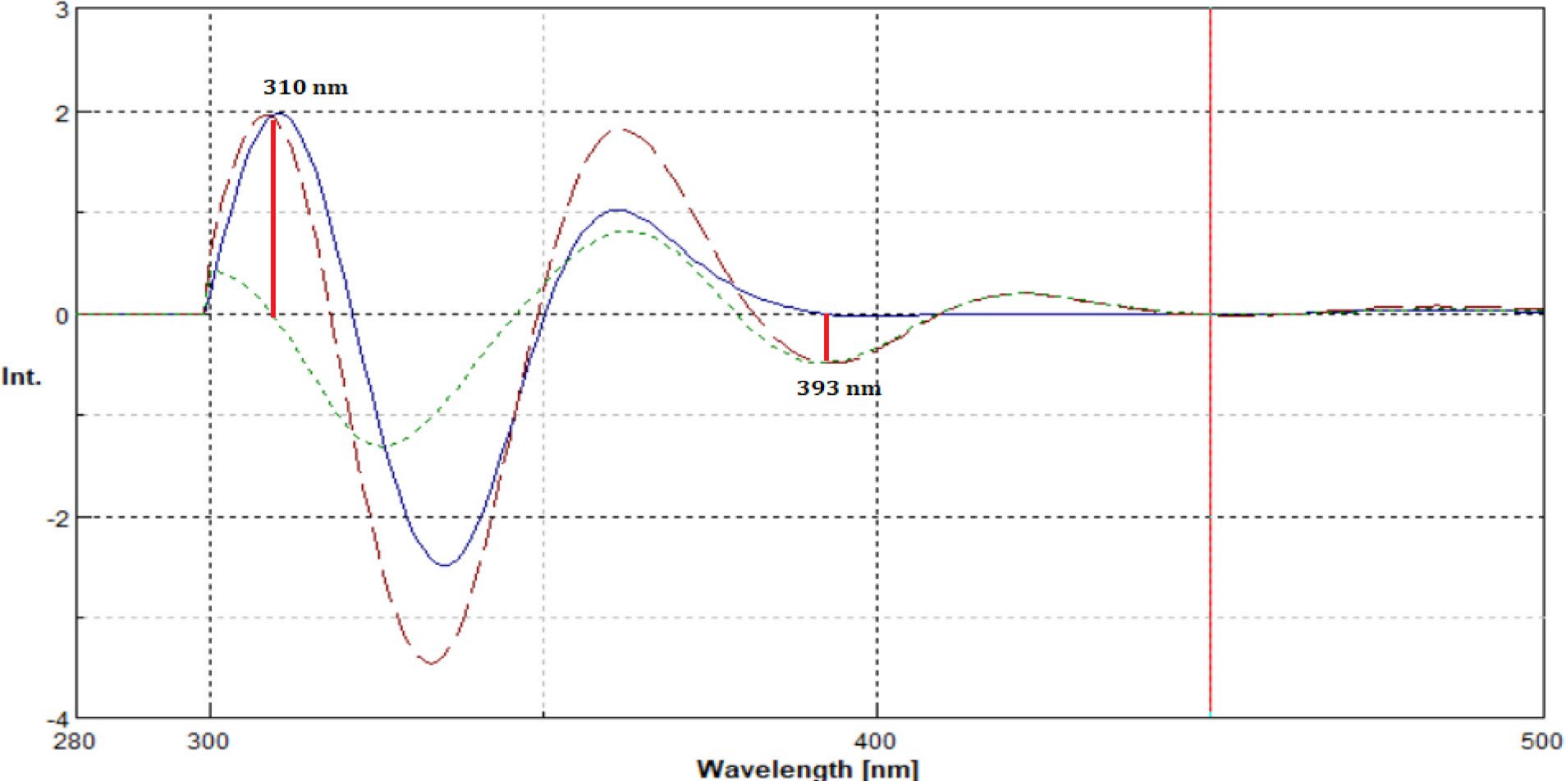
Second-derivative synchronous fluorescence spectra of the mixture containing SIT (2.8 µg/ml) and EMP (0.9 µg/ml) (^__ __^), EMP (0.9 µg/ml) (^___^), and SIT (2.8 µg/ml) (---) at Δ = 60 nm.

The selectivity of the proposed method was further verified through the analysis of synthetic mixtures containing both drugs, where clear discrimination between EMP and SIT signals was achieved (Fig. 6). In addition, successful application to pharmaceutical formulations demonstrated the absence of interference from common excipients (Fig. S1), confirming its suitability for routine quality control analysis. The method was also effectively applied to spiked human plasma samples, where no significant interference from endogenous matrix components was observed (Fig. S2), highlighting its applicability to biological analysis.

Overall, the combination of synchronous fluorescence spectroscopy with second-derivative transformation provides an efficient approach for resolving the spectral overlap of EMP and SIT, enabling their simultaneous determination with high selectivity and sensitivity without the need for prior separation.

### Optimization of experimental parameters

To achieve optimal sensitivity, selectivity, and spectral resolution, the experimental variables influencing the synchronous fluorescence behaviour of empagliflozin and sitagliptin were systematically optimized. Each parameter was evaluated independently while keeping all other conditions constant. The factors investigated included the synchronous wavelength interval (Δλ), organized media, pH, buffer system, and diluent.

The synchronous wavelength interval (Δλ) is a critical parameter governing both fluorescence intensity and spectral resolution. Therefore, Δλ values ranging from 20 to 120 nm were examined. At lower Δλ values (< 40 nm), weak fluorescence signals accompanied by partial spectral overlap were observed. Increasing Δλ resulted in enhanced signal intensity and improved peak separation, with Δλ = 60 nm yielding sharp, symmetrical, and well-resolved peaks for both analytes. Further increases beyond 80 nm led to peak broadening and a gradual decline in fluorescence intensity. Accordingly, Δλ = 60 nm was selected as the optimal value for subsequent measurements.

**Fig. 7:**
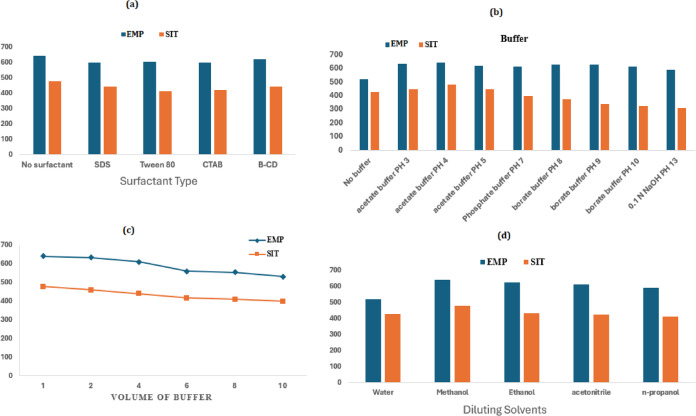
Optimization of experimental conditions for EMP and SIT, including surfactant effect (**a**), buffer type (**b**), buffer volume (**c**), and diluting solvent (**d**).

The influence of organized media was assessed using various surfactants, including β-cyclodextrin, carboxymethylcellulose, Tween 80, cetrimide, and sodium dodecyl sulfate (SDS). None of the tested surfactants produced a significant increase in fluorescence intensity (Fig. 7a), indicating the absence of favorable micellar or inclusion effects under the conditions studied.

The effect of pH was investigated using acetate, phosphate, and borate buffer systems. SIT exhibited a pronounced pH dependence, showing enhanced and more stable fluorescence under mildly acidic conditions, likely due to protonation of its amino groups and suppression of photoinduced electron transfer. In contrast, EMP fluorescence remained relatively stable across the pH range studied, with slight instability observed under alkaline conditions. Among the tested systems, acetate buffer (0.1 M, pH 4.0) provided the most favorable analytical performance, offering enhanced sitagliptin fluorescence, stable EMP response, and minimal background interference (Fig. 7b and c).

The effect of different diluting solvents, including water, ethanol, methanol, acetonitrile, and *n*-propanol, was investigated. Methanol provided more consistent and reproducible fluorescence signals, along with well-defined synchronous spectral profiles. In addition, methanol offers the advantage of acting as a protein-precipitating agent, facilitating its use in plasma sample preparation and ensuring methodological consistency between pharmaceutical and biological analyses. Therefore, methanol was selected as the diluent for its overall suitability in terms of signal stability, procedural simplicity, and compatibility with diverse sample matrices, while minimising solvent consumption (Fig. 7d).

## Method validation

According to ICH guidelines 55, the proposed method was successfully validated for accuracy, precision, selectivity, linearity, limit of detection, and limit of quantification, ensuring its reliability and suitability for pharmaceutical analysis and spiked-plasma applications.

### Linearity and range

When the second-derivative synchronous fluorescence (^2^D) peak amplitude was plotted against drug concentration, a linear relationship was obtained for EMP over the range of 0.040–1.2 µg/ml at 310 nm, and for SIT over the range of 0.1–4 µg/ml at 393 nm. Linear regression analysis yielded the following equations:

^2^ D = 0.0022 C − 0.0184 (*r* = 0.9996) for empagliflozin at 310 nm.

^2^ D = −0.172 C + 0.001 (*r* = 0.9998) for sitagliptin at 393 nm,

where ^2^D represents the SDSFS peak amplitude, C is the drug concentration (µg/ml), and r is the correlation coefficient. The high correlation coefficients, together with the low standard deviations of the slope and intercept, confirm excellent linearity and reliability across the studied ranges (Table 1). Residual analysis showed a random distribution around the regression line, indicating the absence of systematic error. Moreover, analysis of variance (ANOVA) demonstrated that the regression was statistically significant (*p* < 0.05), confirming the model’s suitability for quantitative analysis.

**Table 1:** Regression and validation data for the assessment of EMP and SIT using the proposed method.

Parameters	EMP	SIT
Wavelength (nm)	310 nm	393 nm
Linearity range (µg/ml)	0.04–1.2 µg/ml	0.1–4 µg/ml
Determination coefficient (r^2^)	0.9996	0.9998
Intercept	−0.0184	0.001
Slope	0.0022	−0.172
LOD (µg/mL)	0.011 µg/ml	0.031 µg/ml
LOQ (µg/mL)	0.033 µg/ml	0.0.93 µg/ml
Accuracy (%R) ^a^	98.43	99.51
Precision (%RSD) ^b^ -Repeatability -Intermediate precision	0.886 1.101	0.972 1.331
Robustness (%RSD) - Δλ (± 1 nm) - pH (± 0.1) - buffer volume (acetate buffer) (± 0.1 ml)	1.1 0.983 1.412	1.271 0.967 0.794

^a^ Average of nine determinations (three concentrations repeated three times).

^b^ RSD of nine determinations (three concentrations repeated three times).

### Limit of detection and quantification

According to ICH Q2(R1) guidelines, the limits of detection (LOD) and quantification (LOQ) were calculated using the following equations:

LOD = 3.3σ/S.

LOQ = 10σ/S.

where S is the slope of the calibration curve and σ is the standard deviation of the intercept. The obtained values (Table 1) indicate high method sensitivity, with LODs of 0.011 µg/ml and 0.031 µg/ml for EMP and SIT, respectively, and corresponding LOQs of 0.033 µg/ml and 0.093 µg/ml.

### Accuracy and precision

The accuracy of the proposed method was evaluated through recovery studies performed on EMP and SIT in pharmaceutical formulations and spiked human plasma at three concentration levels (low, medium, and high) within the linearity range, in accordance with ICH Q2(R1) guidelines (Table S1). The obtained recoveries ranged from 98.0 to 102.0% for pharmaceutical formulations and 96.0–104.0% for plasma samples. These results confirm the accuracy of the method and its suitability for analyzing both dosage forms and complex biological matrices.

Precision was assessed at the same concentration levels. Intra-day precision (repeatability) was evaluated by analyzing three replicates within a single day, while inter-day precision (intermediate precision) was assessed over three consecutive days. Precision was expressed as %RSD for each level, with values below 2.0% for both analytes (Table 1), demonstrating excellent precision and reproducibility.

### Specificity and selectivity

The specificity of the proposed method was evaluated using laboratory-prepared mixtures (Table 2), pharmaceutical formulations, blank plasma, and spiked plasma samples. No interference from excipients or endogenous plasma components was observed, confirming the method’s high selectivity.

**Table 2:** EMP and SIT assessment in laboratory-prepared mixtures using the proposed technique.

Added EMP (µg/mL)	EMP found (µg/mL)	EMP (%*R*)	Added SIT (µg/mL)	SIT found (µg/mL)	SIT (%*R*)
0.2	0.196	98.0	0.8	0.81	101.3
0.4	0.406	101.5	1.6	1.57	98.1
0.6	0.607	101.2	2.4	2.43	101.3
0.8	0.794	99.3	3.2	3.18	99.4
1	0.982	98.2	4	4.04	101.0
Mean ± %RSD 99.62 ± 1.636			Mean ± %RSD 100.20 ± 1.399		

The standard addition technique was further applied (Table 3), and the obtained recoveries were within acceptable limits, indicating that excipients and plasma proteins did not affect analyte determination.

**Table 3:** Quantitative determination of EMP and SIT using the proposed method and standard addition technique.

Drug	Pharmaceutical formulation taken (µg/ml)	Pharmaceutical formulation found^a^ (µg/ml)	Pure added (µg/ml)	Pure found^b^ (µg/ml)	Pure recovery (%*R*)
EMP	25	24.61	0.2	0.203	101.50
			0.4	0.395	98.75
			0.6	0.589	98.17
			0.8	0.81	101.25
	Mean ± %RSD				99.92 ± 1.704
SIT	100	100.83	0.8	0.794	99.25
			1.6	1.62	101.25
			2.4	2.37	98.75
			3.2	3.14	98.125
	Mean ± %RSD				99.34 ± 1.352

^a^ Average of five determinations.

^b^ Average of three determinations.

Matrix effects were further assessed by comparing responses obtained from spiked plasma samples (Table 5) with those from calibration standards using Student’s *t*-test and *F*-test at a 95% confidence level. No significant differences were observed, confirming negligible matrix effects and the absence of signal suppression or enhancement.

### Statistical comparison with the reported method

To validate the performance of the proposed method, the results were statistically compared using Student’s T-test and F-test at a 95% confidence level.

The calculated t and F values were lower than the theoretical values at a 95% confidence level, indicating no significant difference in accuracy and precision between the proposed and reported methods (Table 4).

**Table 4:** Assessment of EMP and SIT in Gliempaflozin SITA^®^ tablets by the proposed method with statistical comparison to the reported method.

Parameters	Proposed method		Reported method^[49]^	
	EMP	SIT	EMP	SIT
Number of measurements	5	5	5	5
Mean % recovery ^a^	99.33	99.74	100.11	99.96
% RSD	1.472	0.971	1.181	0.631
Variance	2.167	0.943	1.395	0.398
Students’ *T*-test ^b^	1.294 (2.306)	1.628	**——**	**——**
*F*-value ^b^	1.553 (6.388)	2.369	**——**	**——**

^a^ Average of three determinations.

^b^ The values in parentheses are tabulated values of t and F at(*P*=0.05).

### Robustness

The robustness of the proposed spectrofluorimetric method was investigated by slightly varying the optimal conditions. For example, the pH of a 0.5 M acetic acid solution was changed by ± 0.1 pH units, the volume of the acidic solution was changed by ± 0.1 ml, and the wavelength (Δλ) was altered by ±1 nm while keeping all other conditions unchanged. It was found that there were no statistically significant changes in the results, as indicated by %Recovery values within acceptable limits and %RSD values below 2% (Table 1). Therefore, the proposed method is robust, reliable, and efficient for determining EMP and SIT.

### Pharmaceutical tablets application

The proposed method was successfully applied to the analysis of EMP and SIT in pharmaceutical formulations using a pharmaceutical calibration curve that minimizes excipient interference.

The results were in good agreement with the labeled claims, and standard addition confirmed the absence of interference from excipients (Table 3).

### Application to spiked human plasma

Owing to its high sensitivity and low detection limits, the proposed method was successfully applied to the determination of EMP and SIT in spiked human plasma samples using a plasma-based calibration curve. Blank plasma samples confirmed the absence of endogenous interference. Furthermore, a comparison between calibration in a pure solvent and in a plasma matrix indicated no significant signal suppression or enhancement, confirming negligible matrix effects.

According to population pharmacokinetic studies, the mean maximum plasma concentrations of SIT and EMP are approximately 370 ng/mL and 667 ng/mL, respectively^68–70^, which fall within the established linearity ranges.

The results demonstrate accurate and precise quantification, with mean recoveries of 97.49 ± 0.66% for EMP and 98.07 ± 0.61% for SIT (Table 5). The low %RSD values indicate excellent precision, and no significant interference from plasma components was observed.

**Table 5:** Recovery data of EMP and SIT from spiked human plasma using the proposed method.

Try	EMP added amount (ng/mL)	EMP found amount (ng/mL)	SIT added amount (ng/mL)	SIT found amount (ng/mL)	EMP (%*R*) ^a^	SIT (%*R*) ^a^
1	50	48.52	100	98.52	97.03	98.52
2	75	72.83	150	146.79	97.11	97.86
3	100	98.56	200	197.22	98.56	98.61
4	125	121.40	250	245.625	97.12	98.25
5	150	146.42	300	291.36	97.61	97.12
Mean ± % RSD					97.49 ± 0.66	98.07 ± 0.61

^a^ Average of three determinations.

### Comparison with previously reported methods

To evaluate the analytical performance of the proposed method, a comprehensive comparison was conducted with previously reported methods for the determination of EMP and SIT ^**(48–49)**^. As summarized in Table S2, the developed second-derivative synchronous fluorescence method demonstrates several notable advantages over both reported HPLC and UV-spectrophotometric approaches.

Compared with reported HPLC methods, the proposed method offers a significantly shorter analysis time (approximately 2 min versus 3–6 min) and reduced consumption of organic solvents, while maintaining comparable or superior sensitivity. The achieved limits of detection (LOD) were 0.031 µg/ml for SIT and 0.011 µg/ml for EMP, which are markedly lower than those reported for HPLC (1.63 µg/ml for SIT and 0.11 µg/ml for EMP), highlighting the enhanced sensitivity of the proposed approach.

Compared with the reported UV-spectrophotometric method, although both methods have similar analysis times and simple sample preparation, the proposed method demonstrates superior sensitivity and selectivity. The UV method shows relatively higher detection limits (LOD > 1 µg/ml) and moderate selectivity due to spectral overlap between EMP and SIT, which may necessitate additional mathematical manipulation or compromise accuracy in complex matrices. In contrast, the second-derivative synchronous fluorescence technique provides excellent spectral resolution and selectivity, enabling accurate simultaneous determination without interference.

A key advantage of the current method is its applicability across different matrices, including pharmaceutical formulations and human plasma, with satisfactory recoveries and negligible matrix interference. The sample preparation procedure is straightforward, requiring only simple protein precipitation for plasma samples, thereby enhancing its suitability for routine analytical applications.

Furthermore, the method does not rely on specialized nanomaterials or sophisticated instrumentation, increasing its accessibility to standard analytical laboratories. Collectively, the method combines high sensitivity, rapid analysis, operational simplicity, and reduced environmental impact, making it a practical and sustainable alternative for routine analysis.

### Greenness, blueness, and whiteness assessment

The environmental, practical, and analytical performance of the proposed method was comprehensively evaluated using multiple sustainability assessment tools, including NEMI, Analytical Eco-Scale, Complex MoGAPI, AGREEprep, CaFRI, BAGI, and the RGB 12 algorithm^71–74^**(**Table 6) This multi-metric approach ensures a holistic evaluation of greenness, blueness, and whiteness.

**Table 6:** Comprehensive evaluation of greenness, blueness, and whiteness for the proposed method.

	EMP and SIT determination in pure standard solutions	EMP and SIT determination in plasma samples
NEMI tool		
ComplexMoGAPI tool		
AGREE prep tool		
CaFRI tool		
BAGI tool		
RGB 12 algorithm		

### NEMI (greenness)

The NEMI pictograms confirm full compliance with green chemistry principles, as all four quadrants are green. This can be attributed to the absence of hazardous reagents, minimal solvent consumption, and the elimination of multi-step extraction procedures. In contrast, HPLC methods typically require larger volumes of organic solvents (e.g., acetonitrile or methanol in the mobile phase), while UV methods may involve additional reagents or sample treatment steps, slightly reducing their greenness profiles.

### Analytical eco-scale (greenness)

The proposed method achieved an Eco-Scale score of 89, classifying it as an excellent green analytical procedure. This high score is primarily attributed to low solvent consumption, the absence of derivatization steps, and reduced energy requirements. In comparison, HPLC methods (score: 69) incur penalties for high solvent consumption, waste generation, and energy-intensive instrumentation, whereas UV methods (score: 82) incur moderate penalties due to lower selectivity and the potential need for additional data processing.

### Complex MoGAPI (greenness)

The Complex MoGAPI assessment demonstrated high greenness scores (83 for pure standards and 79 for plasma), reflecting environmentally favorable analytical procedures. The relatively lower scores observed for HPLC (66) are mainly associated with solvent-intensive chromatographic operations and increased waste generation, whereas UV methods (79) are affected by moderate analytical performance and selectivity limitations.

### AGREEprep (greenness)

AGREEprep scores (0.80 for standards and 0.76 for plasma) indicate strong compliance with green sample preparation principles. This is mainly due to the use of a simple protein-precipitation step that requires minimal solvent and handling. In contrast, HPLC methods (0.62) often involve more complex preparation and filtration steps, while UV methods (0.72) may require additional dilution or sample manipulation.

### CaFRI (greenness)

The CaFRI scores (85 and 78) reflect a reduced carbon footprint, mainly due to shorter analysis time and the avoidance of high-pressure systems. HPLC methods (60) exhibit a higher environmental impact due to continuous energy consumption, solvent preparation, and instrument operation, whereas UV methods (76) show moderate performance with lower energy demand but reduced analytical efficiency.

### BAGI (blueness)

The BAGI score (87.5) highlights the excellent practical efficiency of the proposed method, driven by rapid analysis, minimal reagent consumption, and operational simplicity. Lower scores for HPLC (72.5) are associated with longer run times, system maintenance, and operational complexity, while UV methods (75) are limited by lower sensitivity and selectivity.

### RGB 12 algorithm (whiteness)

The RGB 12 assessment demonstrated excellent overall performance, with whiteness scores of 94.9 for standards and 91.4 for plasma. These high scores result from the combination of superior analytical performance (high sensitivity and selectivity), strong environmental sustainability, and operational simplicity. In comparison, HPLC methods (81) are constrained by environmental and operational factors, whereas UV methods (84) are primarily limited by lower analytical performance, despite their acceptable greenness.

### Overall assessment

Collectively, the proposed method demonstrates a superior balance between greenness, blueness, and whiteness. Its enhanced sustainability profile is primarily attributed to minimal solvent consumption, simplified sample preparation, reduced energy requirements, and improved analytical performance.

In contrast, the comparatively lower scores of HPLC methods stem from their greater environmental burden and operational complexity, whereas UV spectrophotometric methods are primarily limited by insufficient sensitivity and selectivity.

Accordingly, the proposed method represents a highly efficient, sensitive, and environmentally sustainable approach for the routine simultaneous determination of EMP and SIT.

### Limitation

Although spectral resolution is significantly improved by the second-derivative transformation at Δλ = 60 nm, the derivative process may increase instrumental noise and reduce the signal-to-noise ratio, particularly at low analyte concentrations. As a result, maintaining sensitivity and accuracy requires careful adjustment of the instrument settings.

## Conclusion

A highly selective, sensitive, and environmentally sustainable second-derivative synchronous spectrofluorimetric method was successfully developed for the simultaneous determination of EMP and SIT in pharmaceutical formulations and spiked human plasma. Complete spectral resolution was achieved at Δλ = 60 nm via second-derivative processing, effectively overcoming the inherent spectral overlap between the two analytes without prior separation.

The method demonstrated excellent analytical performance, including outstanding linearity, accuracy, precision, and robustness, in full compliance with ICH validation guidelines. High sensitivity was achieved, with LODs of 0.011 µg/ml for empagliflozin and 0.031 µg/ml for sitagliptin, and LOQs of 0.033 µg/ml and 0.093 µg/ml, respectively, enabling reliable trace-level determination in complex matrices.

The simplicity of the procedure, based on single-step protein precipitation of plasma samples and minimal solvent use, enhances its practicality, safety, and suitability for routine applications. Furthermore, the method exhibited excellent environmental performance, as confirmed by a comprehensive multi-tool assessment (AGREEprep, NEMI, the Analytical Eco-Scale, Complex MoGAPI, CaFRI, BAGI, and RGB 12). This evaluation demonstrated a significantly reduced environmental impact and carbon footprint compared to reported techniques, while also highlighting superior analytical performance and practical applicability.

Overall, the proposed method provides a reliable, cost-effective, and eco-friendly analytical approach, combining high sensitivity, operational simplicity, and strong analytical performance, making it well-suited for routine quality control and bioanalytical applications.

## Supplementary Information

Below is the link to the electronic supplementary material.

Supplementary Material 1

## Data Availability

The data and materials used in this study are available on reasonable request.
