# Supplementary material for: Eco-friendly second-derivative synchronous fluorescence method for the determination of empagliflozin and sitagliptin in tablets and plasma samples
Source: Sci Rep. 2026 May 20;16:15708. doi: 10.1038/s41598-026-53178-z (PMC13190686; doi:10.1038/s41598-026-53178-z)
Supplement: Supplementary file 1 — Supplementary Material 1 [file 41598_2026_53178_MOESM1_ESM.docx]

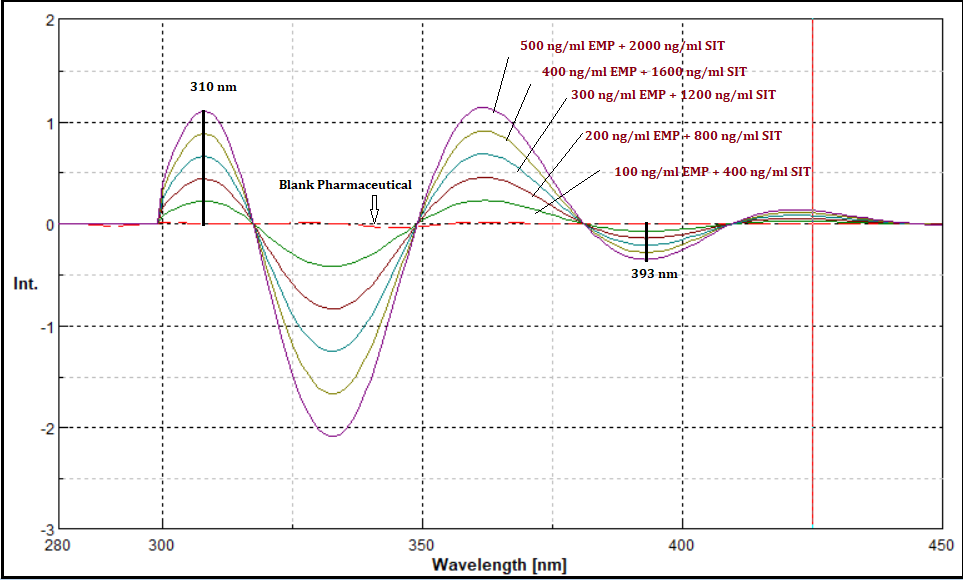


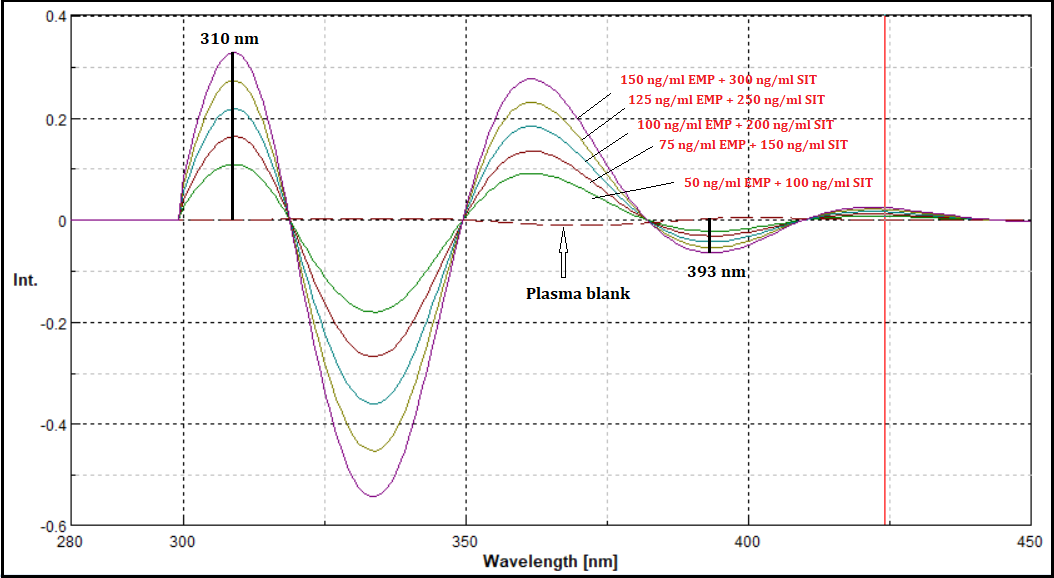
Fig. S1 presents the second-derivative synchronous fluorescence spectra of SIT and EMP in a pharmaceutical application and of the excipient blank at Δ = 60 nm.

Fig. S2 presents the second-derivative synchronous fluorescence spectra of SIT and EMP in plasma and blank plasma at Δ = 60 nm

Table S1: Recovery and precision data of EMP and SIT using the proposed method.

| Empagliflozin | | | | |
| --- | --- | --- | --- | --- |
| Conc Level | Concentration (µg/ml) | Recovery (%R) | (%RSD) (Repeatability) | (%RSD) (Intermediate Precision) |
| Low | 0.05 | 98.33 | 0.876 | 0.978 |
| Mid | 0.6 | 98.95 | 0.883 | 1.037 |
| High | 1.2 | 98.02 | 0.899 | 1.292 |
| Average | | 98.43 | 0.886 | 1.101 |
| Sitagliptin | | | | |
| Conc Level | Concentration (µg/ml) | Recovery (%R) | (%RSD) (Repeatability) | (%RSD) (Intermediate Precision) |
| Low | 0.1 | 98.42 | 1.112 | 1.447 |
| Mid | 2 | 99.78 | 0.933 | 1.290 |
| High | 4 | 100.33 | 0.872 | 1.257 |
| Average | | 99.51 | 0.972 | 1.331 |

Table S2: Comparative analytical performance and greenness evaluation of the proposed method versus reported HPLC and UV-spectrophotometric methods.

| Parameter | Proposed Method | Reported HPLC Method | Reported UV- Spectrophotometric Method |
| --- | --- | --- | --- |
| LOD (µg/ml) | 0.011 / 0.031 | 0.11 / 1.63 | >1 |
| LOQ (µg/ml) | 0.033 / 0.093 | 0.35 / 4.92 | >1 |
| Analysis time | 2 min | 3–6 min | 2 min |
| Selectivity | Excellent | Excellent | Moderate |
| Sample preparation | Simple | Complex | Simple |
| Eco-Scale | 89 | 69 | 82 |
| Complex MoGAPI | 83 | 66 | 79 |
| AGREEprep | 0.8 | 0.62 | 0.72 |
| BAGI | 87.5 | 72.5 | 75 |
| CaFRI | 85 | 60 | 76 |
| RGB 12 | 94.9 | 81 | 84 |
